# Nirsevimab reduced acute otitis media infections in infants—a nationwide interrupted time-series analysis

**DOI:** 10.1007/s00431-026-07508-y

**Published:** 2026-10-09

**Authors:** Niko Leskinen, Marjo Renko, Ilari Kuitunen

**Affiliations:** 1 Institute of Clinical Medicine, University of Eastern Finland, P.O. Box 1627, 70211 Kuopio, Finland; 2 Department of Pediatrics, Kuopio University Hospital, Kuopio, Finland

**Keywords:** RSV, Nirsevimab, Acute otitis media, Infants, Prophylaxis

## Abstract

Respiratory syncytial virus (RSV) is a major cause of acute respiratory tract infections in young children worldwide. Acute otitis media (AOM) is among the most frequent complications of RSV infection and the leading indication for antibiotic prescribing in this age group. The aim of this study was to evaluate the effectiveness of nirsevimab in reducing the incidence of AOM among infants. We conducted a nationwide register-based interrupted time-series study using data on live births, laboratory-confirmed RSV detections, and AOM diagnoses from four consecutive RSV seasons (2022–2023 to 2025–2026). Monthly infant populations at risk were reconstructed from birth cohorts to estimate incidence rates during the peak RSV seasons (December–March) which were compared between two pre-implementation and two post-implementation RSV seasons in infants aged < 12 months, stratified into age groups of 0–2, 3–5, and 6–11 months. The study population consisted of 181,999 infants and the follow-up consisted of 2,022,261 infant-months. A total of 3699 RSV-positive findings and 15,830 AOM diagnoses were included in the primary analysis. Following nirsevimab implementation, the incidence of AOM and RSV among infants aged < 12 months decreased by 44% (RR 0.56, 95% CI 0.54–0.58) and 83% (RR 0.17, 95% CI 0.15–0.18), respectively. The reduction was greatest in the youngest age (0–2 months) group.

*Conclusion*: Implementation of a nirsevimab immunisation programme was associated with a substantial reduction in AOM incidence among infants aged < 12 months, suggesting that RSV immunisation may reduce the burden of AOM in early childhood.

**Table:** 

**What's Known**: • *RSV is a major cause of morbidity during infancy, and acute otitis media is among its most frequent complications.* • *Nirsevimab has demonstrated high efficacy in preventing severe RSV disease in young children; however, its effect on outpatient RSV-related complications has not been well established.* **What is New:** • *This study provides population-based real-world evidence on the impact of nirsevimab on acute otitis media.* • *Following the implementation of nirsevimab prophylaxis, the incidence of acute otitis media among infants decreased significantly.*

**Supplementary Information:**

The online version contains supplementary material available at https://doi.org/10.1007/s00431-026-07508-y.

## Introduction

Respiratory syncytial virus (RSV) is one of the leading causes of respiratory viral infections among children [1]. In 2019, RSV was associated with an estimated 33 million lower respiratory tract infections and over 3.5 million hospital admissions worldwide, highlighting its substantial global burden [2]. As infants younger than 6 months account for most RSV-associated hospitalizations and deaths, young age, together with underlying medical conditions, remains the primary risk factor for severe RSV infection [3, 4]. In recent years, the development and clinical implementation of various immunisation strategies have played a crucial role in reducing the burden of RSV, especially in high-income countries [5].

Both nirsevimab, a long-acting monoclonal antibody, and maternal RSV vaccination are highly effective in preventing severe RSV infection among infants during their first RSV season, with the recently approved clesrovimab also demonstrating high efficacy in preventing RSV-associated lower respiratory tract disease [6–8]. Nirsevimab is currently more widely used globally despite its higher cost, as it may provide somewhat better protection than maternal RSV vaccination and is supported by a more extensive and mature evidence base [9–11]. However, the efficacy of nirsevimab prophylaxis in reducing outpatient RSV complications remains largely unknown.

In addition to lower respiratory tract disease, RSV and other viral respiratory infections are frequently associated with AOM, which is highly prevalent in early childhood [12, 13]. During infancy, 20% to 60% of children experience at least one episode of AOM, and by the age of 3 years, this proportion exceeds 80% [14, 15]. Given its high incidence and its status as one of the leading causes of antimicrobial prescribing in children, AOM represents a substantial clinical and economic burden [16, 17].

This study aims to evaluate the effect of nirsevimab prophylaxis on the incidence of AOM in infants under 1 year of age, using nationwide register data from Finland.

## Methods

We conducted a nationwide, register-based interrupted time-series study using aggregated, anonymous statistics compiled by the Finnish Institute for Health and Welfare (THL) under the data-permit authority of Findata (decision THL/540/14.01.00/2026). Three national registers were combined at population level: the Finnish Medical Birth Register (monthly live births), the National Infectious Diseases Register (laboratory-confirmed RSV detections), and the Register of Primary Health Care Visits (AvoHILMO; outpatient acute otitis media (AOM) visits coded ICD-10 H66.0 or H66.9). Monthly counts of RSV detections and AOM visits in infants aged < 12 months were obtained for January 2022–March 2026 and stratified into age bands of 0–2, 3–5, and 6–11 months; the amount of monthly live births was available for January 2022–December 2025. Because the data were aggregate and non-identifiable, the study required no ethics-committee review or individual consent.

Finland introduced universal infant nirsevimab immunisation for the 2024–2025 RSV season (autumn 2024, most places started in October 2024), administered at maternity hospitals to infants born before and during the epidemic and to those aged < 3 months at its onset. So practically, the nirsevimab has been administered to all neonates born from October to April, and any infant aged less than 3 months of age during this window has also been eligible to receive the prophylaxis. As the winter is the peak RSV season in the Northern Hemisphere, we therefore defined epidemic seasons (July–June) 2022–2023 and 2023–2024 as pre-implementation and 2024–2025 and 2025–2026 as post-implementation.

To estimate incidence, we reconstructed the monthly infant population at risk in each age band from rolling birth cohorts (for example, the 0–2-month band in month m comprised live births in months m to m − 2), yielding infant-months of observation. Because official live-birth data were available only through December 2025, live births for January–March 2026 were estimated using the mean monthly number of births during the preceding 12 months (January–December 2025) for reconstruction of infant-month denominators in the 0–2-month age group. The primary analysis compared pre- and post-implementation incidence over the core epidemic window (December–March). We calculated incidence per 1000 infant-months and rate ratios (RRs, post- versus pre-programme) with exact (Poisson) 95% confidence intervals (CIs), separately for RSV detections and AOM diagnoses and by age band. As a sensitivity analysis, we fitted negative-binomial regression to monthly counts across all months, including a post-implementation indicator, age band, and their interaction, first-order harmonic seasonal terms, and log infant-months as an offset. Analyses were performed in Python 3 (statsmodels, scipy).

## Results

**Fig. 1:**
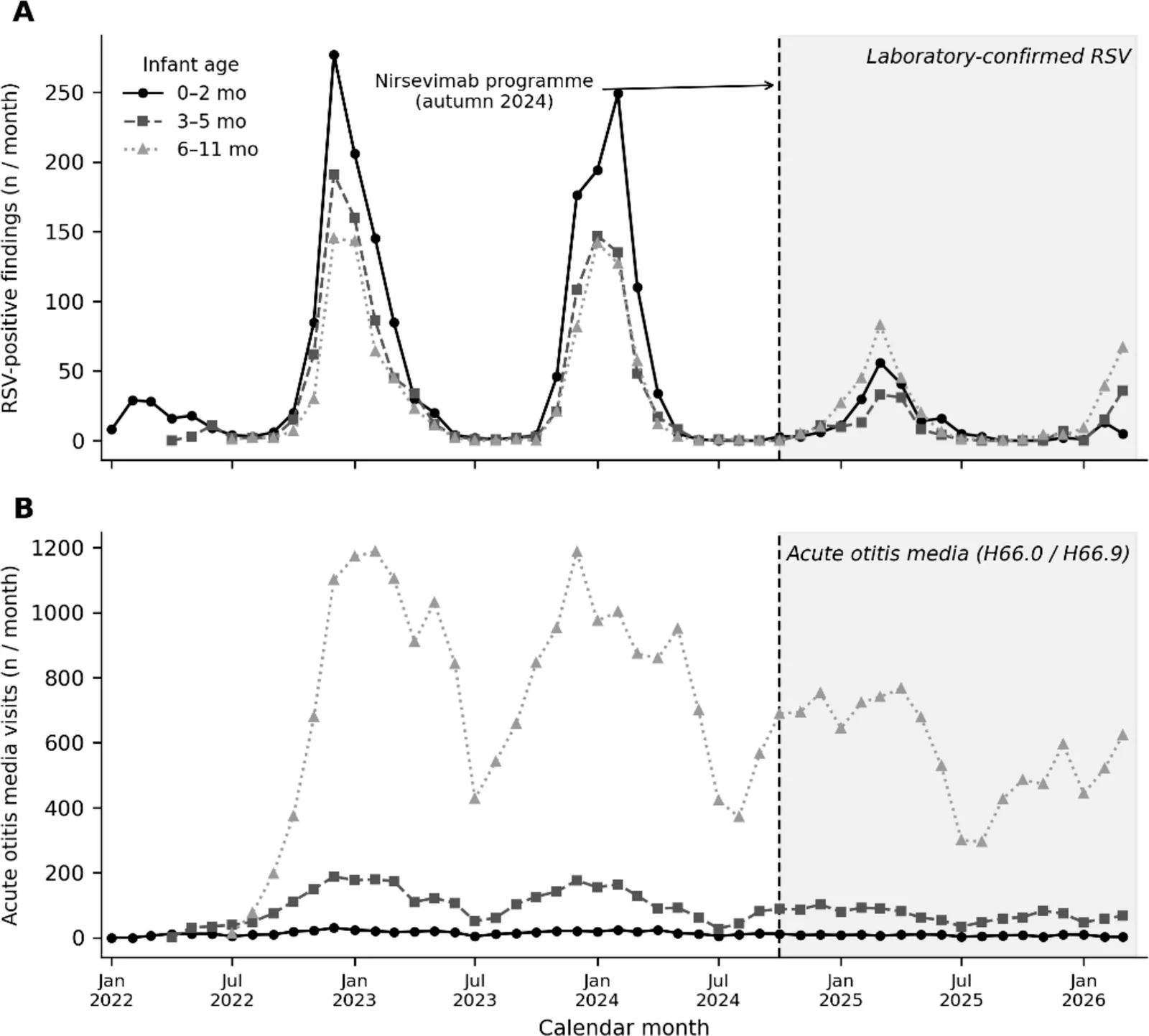
Monthly number of **A** RSV-positive findings and **B** AOM visits among infants

Overall, the study population comprised 181,999 live births and contributed 2,022,261 infant-months at risk. During the primary-analysis epidemic windows (December–March), the pre-implementation seasons contributed 353,641 infant-months at risk (84,530 aged 0–2 months, 90,385 aged 3–5 months, and 178,726 aged 6–11 months), whereas the post-implementation seasons contributed 359,512 infant-months at risk (87,720 aged 0–2 months, 91,038 aged 3–5 months, and 180,754 aged 6–11 months) (Table S1). During the study period, a total of 4607 RSV detections and 35,334 AOM diagnoses were identified. Of these, 3699 RSV detections and 15,830 AOM diagnoses occurred during the core epidemic windows (December–March) across the four study seasons (2022–2023 to 2025–2026). Monthly trends in laboratory-confirmed RSV detections and AOM diagnoses by age group are shown in Fig. 1.

Among infants aged < 12 months, the incidence of laboratory-confirmed RSV detections decreased from 8.95 to 1.49 per 1000 infant-months at risk in the post-implementation period compared with the pre-implementation period, corresponding to an 83% reduction (RR 0.17, 95% CI 0.15–0.18). The reduction was greatest among infants aged 0–2 months (RR 0.08, CI 0.07–0.10) and smallest among those aged 6–11 months (RR 0.35, CI 0.30–0.40) (Table 1).

**Table 1:** Pre- versus post-implementation incidence of laboratory-confirmed RSV and acute otitis media in infants aged < 12 months, by age group, Finland, core epidemic windows (December–March) of the 2022–2023/2023–2024 (pre) and 2024–2025/2025–2026 (post) seasons

Age group	Pre *n*	Pre rate	Post *n*	Post rate	Rate ratio (95% CI)
***Laboratory-confirmed RSV***					
0–2 mo	1442	17.06	124	1.43	0.08 (0.07–0.10)
3–5 mo	920	10.18	125	1.37	0.13 (0.11–0.16)
6–11 mo	804	4.50	284	1.57	0.35 (0.30–0.40)
All < 12 mo	3166	8.95	533	1.49	0.17 (0.15–0.18)
***Acute otitis media***					
0–2 mo	174	2.06	56	0.65	0.31 (0.23–0.43)
3–5 mo	1342	14.85	611	6.71	0.45 (0.41–0.50)
6–11 mo	8603	48.14	5044	27.91	0.58 (0.56–0.60)
All < 12 mo	10,119	28.61	5711	15.93	0.56 (0.54–0.58)

The incidence of AOM decreased from 28.61 to 15.93 per 1000 infant-months at risk in the post-implementation period compared with the pre-implementation period, corresponding to a 44% reduction (RR 0.56, CI 0.54–0.58). The largest reduction was observed among infants aged 0–2 months (RR 0.31, CI 0.23–0.43), followed by those aged 3–5 months (RR 0.45, CI 0.41–0.50). The smallest reduction was observed among infants aged 6–11 months (RR 0.58, CI 0.56–0.60) (Table 1).

## Discussion

This study evaluated the effectiveness of implementation of nirsevimab prophylaxis in reducing the incidence of acute otitis media (AOM) among infants. Following programme implementation, AOM incidence decreased substantially by 44% compared with the pre-programme period (Table 1). The greatest reduction was observed among infants aged 0–2 months, which may be related to the particularly high burden of RSV infection in this age group [2, 18–20]. Unexpectedly, a substantial reduction in both RSV and AOM incidence was also observed among infants aged 6–11 months, despite only a proportion of infants in this age group being eligible for nirsevimab prophylaxis. The reasons for this finding remain uncertain, as individual-level immunisation data were not available. Possible explanations include the catch-up immunisation strategy implemented during programme introduction, resulting in prophylaxis among some older infants, protection extending beyond 6 months in a proportion of recipients, and indirect population-level effects resulting from reduced RSV transmission.

Given that AOM is a common complication of RSV infection and a leading cause of antibiotic prescribing in young children, the substantial reduction in AOM incidence observed in this study suggests that nirsevimab may reduce not only the burden of RSV disease but also its important downstream complications [12, 13, 16, 17]. RSV prophylaxis with nirsevimab may also significantly reduce antibiotic use during infancy, a particularly vulnerable period during which antibiotic exposure has been associated with an increased risk of multiple diseases later in life [21, 22].

The findings of this study are consistent with those of most previous studies, including an interrupted time-series analysis and a test-negative study based on post hoc analyses of data from a prospective study, both of which reported reductions in AOM incidence after the implementation of nirsevimab [23, 24]. One population-based prospective cohort study, however, reported no reduction in AOM rates after nirsevimab implementation compared with historical data from pre-nirsevimab RSV seasons [25]. A lack of harmonised diagnostic criteria and standardised otoscopic validation was suggested as a possible source of bias by the authors.

A major strength of this study is its large-scale, population-based real-world dataset, which covers four consecutive epidemic seasons, more than two million infant-months at risk, and an immunisation coverage of over 90% [26, 27]. As the uptake and knowledge of nirsevimab continue to increase globally, these findings provide valuable information on its population-level impact [28–30]. An important limitation of this study is the lack of individual-level data on nirsevimab administration. As a result, we were unable to directly compare outcomes between infants who did and did not receive nirsevimab. The observed reductions in RSV and AOM incidence should therefore be interpreted as population-level associations following programme implementation rather than estimates of individual-level effectiveness. In addition, changes in healthcare-seeking behaviour, diagnostic practices, or the circulation of other respiratory pathogens between epidemic seasons may have influenced the observed AOM incidence. Furthermore, the magnitude of the observed effect is likely to depend on nirsevimab uptake. The high immunisation coverage achieved in this setting may have contributed to the reductions observed and should be considered when interpreting the findings and comparing them with results from other populations.

## Conclusion

The implementation of RSV prophylaxis with nirsevimab was associated with clear reduction in AOM incidence among infants, with the largest reduction observed in the youngest age groups. Further individual-level studies are needed to assess the effectiveness of nirsevimab in preventing AOM. Future research should also evaluate whether maternal RSV vaccination has similar effects on AOM incidence.

## Supplementary Information

Below is the link to the electronic supplementary material.

ESM 1 (DOCX 13.6 KB)

## Data Availability

Data availeble upon request from the corresponding author.

## Abbreviations

AOM: Acute otitis media
AvoHILMO: Register of Primary Health Care Visits
CI: Confidence interval
ICD-10: International Classification of Diseases, 10th Revision
RR: Rate ratio
RSV: Respiratory syncytial virus
THL: Finnish Institute for Health and Welfare
