# Supplementary material for: Nirsevimab reduced acute otitis media infections in infants—a nationwide interrupted time-series analysis
Source: Eur J Pediatr. 2026 Oct 9;185(11):827. doi: 10.1007/s00431-026-07508-y (PMC13652904; doi:10.1007/s00431-026-07508-y)
Supplement: Supplementary file 1 [file 431_2026_7508_MOESM1_ESM.docx]

Table S1. Infant-months at risk in the pre- and post-programme periods during the primary epidemic window (December–March).

| Age group, months | pre-program | post-program |
| --- | --- | --- |
| 0-2 | 84,530 | 87,720 |
| 3-5 | 90,385 | 91,038 |
| 6-11 | 178,726 | 180,754 |
| total | 353,641 | 359,512 |
